# Mechanical Properties of 3D-Printed Acrylonitrile–Butadiene–Styrene TiO_2_ and ATO Nanocomposites

**DOI:** 10.3390/polym12071589

**Published:** 2020-07-17

**Authors:** Nectarios Vidakis, Markos Petousis, Athena Maniadi, Emmanuel Koudoumas, Marco Liebscher, Lazaros Tzounis

**Affiliations:** 1 Mechanical Engineering Department, Hellenic Mediterranean University, Estavromenos, 71004 Heraklion, Crete, Greece; vidakis@hmu.gr; 2 Department of Materials Science and Technology, University of Crete, Vassilika, Voutes, 70013 Heraklion, Crete, Greece; maniadi@materials.uoc.gr; 3 Center of Materials Technology and Photonics, Hellenic Mediterranean University, Estavromenos, 71004 Heraklion, Crete, Greece; koudoumas@hmu.gr; 4 Institute of Construction Materials, Technische Universität Dresden, DE-01062 Dresden, Germany; 5 Department of Materials Science and Engineering, University of Ioannina, 45110 Ioannina, Greece; latzounis@uoi.gr

**Keywords:** fused filament fabrication (FFF), 3D printing, nanocomposites, flexural strength, tensile strength, acrylonitrile butadiene styrene (ABS), titanium dioxide (TiO_2_), antimony doped tin oxide (ATO)

## Abstract

In order to enhance the mechanical performance of three-dimensional (3D) printed structures fabricated via commercially available fused filament fabrication (FFF) 3D printers, novel nanocomposite filaments were produced herein following a melt mixing process, and further 3D printed and characterized. Titanium Dioxide (TiO_2_) and Antimony (Sb) doped Tin Oxide (SnO_2_) nanoparticles (NPs), hereafter denoted as ATO, were selected as fillers for a polymeric acrylonitrile butadiene styrene (ABS) thermoplastic matrix at various weight % (wt%) concentrations. Tensile and flexural test specimens were 3D printed, according to international standards. It was proven that TiO_2_ filler enhanced the overall tensile strength by 7%, the flexure strength by 12%, and the micro-hardness by 6%, while for the ATO filler, the corresponding values were 9%, 13%, and 6% respectively, compared to unfilled ABS. Atomic force microscopy (AFM) revealed the size of TiO_2_ (40 ± 10 nm) and ATO (52 ± 11 nm) NPs. Raman spectroscopy was performed for the TiO_2_ and ATO NPs as well as for the 3D printed nanocomposites to verify the polymer structure and the incorporated TiO_2_ and ATO nanocrystallites in the polymer matrix. The scope of this work was to fabricate novel nanocomposite filaments using commercially available materials with enhanced overall mechanical properties that industry can benefit from.

## 1. Introduction

In the fused filament fabrication (FFF) 3D printing process, a material in strand form is deposited in layers one after the other to produce 3D structures [1,2]. Different materials have been used in 3D printing, such as sugars [3], thermoplastics and thermoplastic composites [4], photopolymers [5], glass [6], metals [7], metal oxides [8], and ceramics [9], that can be 3D printed into complex shapes. An interesting finding that should be mentioned regarding the 3D printing manufacturing technology is that the mechanical properties of 3D printed ABS parts can differ, depending on the process parameters selected among other factors, in addition to the fact that FFF introduces anisotropy to the final parts [10,11,12,13]. Overall, the ABS polymer mechanical properties in 3D printing have been thoroughly reported in literature [10,14,15,16,17,18].

Nowadays, since a strong demand has emerged for 3D printable materials having additional functionality, as well as improved physical and mechanical properties, the development of tailor-made nanocomposites is of huge interest in engineering science. Specifically, various nanocomposite filaments for 3D printing have received recently a significant interest due to the nanoscale interactions of filler–filler as well as filler-matrix interactions that have profound implications on the macroscopic behavior of the nanocomposite materials, the printing process, the final nanocomposite 3D printed part, etc. Therefore, all dimensions of materials (nano, micro, macro) with a structural character contribute to the overall mechanical performance and durability of the resulting 3D printed part consisting of a nanocomposite filament [4].

For selected nanocomposites, enhancement of functional properties is reported. For instance, the mechanical strength of ABS and PLA reinforced with TiO_2_ nanoparticles (NPs) was found to be improved compared to the unfilled polymer, i.e., virgin ABS and virgin PLA, respectively [19,20,21,22,23,24,25]. TiO_2_ NPs were also found to increase the dielectric properties of polymers for application in insulating materials [26]. Moreover, TiO_2_ nanocomposites can be employed in photocatalytic applications as environment organic pollutants degradants [27,28]. Therefore, ABS-TiO_2_ nanocomposites are envisaged to find various applications, i.e., (i) increase the mechanical performance of the pure polymer thermoplastic matrix, (ii) induce dielectric properties with remarkable and variable responses to electrical fields, and (iii) used as self-cleaning materials due to the pronounced photocatalytic properties of TiO_2_. As such, 3D printing of ABS-TiO_2_ could become a viable way to fabricate custom, on-demand parts that can meet any of these technological needs. Thus, TiO_2_ was selected as one of the fillers in this research work.

Antimony doped tin oxide (ATO) exhibits a great number of functional properties such as resistivity, optical transmission, surface composition and catalytic activity, all depending on the nanoparticle’s preparation technique [29,30]. Regarding the utilization of ATO as filler, it is mostly studied in literature for the dielectric properties and the control of conductivity performance that it can induce in the different polymeric matrices [31,32,33].

In the study at hand, both TiO_2_ and ATO NPs have been investigated as nanofillers in an ABS thermoplastic matrix, where no available literature has been established concerning their effect on the mechanical properties of 3D printed parts, so far. This work focuses on enriching literature by studying the effect of these fillers, on various concentrations, in ABS 3D printed structures. Melt mixing and 3D printing have the advantage to be both completely solvent-free and cost-efficient methods for fabricating high-quality ABS/TiO_2_ and ABS/ATO nanocomposite materials and parts, respectively, and thus both can be directly implemented at an industrial 3D printing manufacturing scale. The nanocomposites studied herein were also analyzed regarding their microstructure and surface morphology using scanning electron microscopy (SEM). Thermogravimetric analysis (TGA) was performed to study the thermal behavior of ABS. Finally, AFM analysis showed the nanoparticle average size, while Raman spectroscopy proved the ABS chemical structure unaltered after the melt mixing and 3D printing processes, as well as the incorporated TiO_2_ and ATO nanocrystallites at different concentrations in the nanocomposite 3D printed materials.

## 2. Materials and Methods

### 2.1. Materials

The polymer matrix used in this work was industrial grade Acrylonitrile Butadiene Styrene (ABS) Terluran Hi-10 in fine powder form purchased from INEOS Styrolution (Frankfurt, Germany). The nanomaterials selected as fillers were the Degussa Evonik P25 Titanium Dioxide (TiO_2_) (Essen, Germany) with average particle size of 25–50 nm and ATO received from Sigma Aldrich (St. Louis, MO, USA) with an average particle size < 50 nm.

### 2.2. Fabrication and 3D Printing of ABS-TiO_2_ and ABS/ATO Nanocomposite Filaments

**Figure 1:**
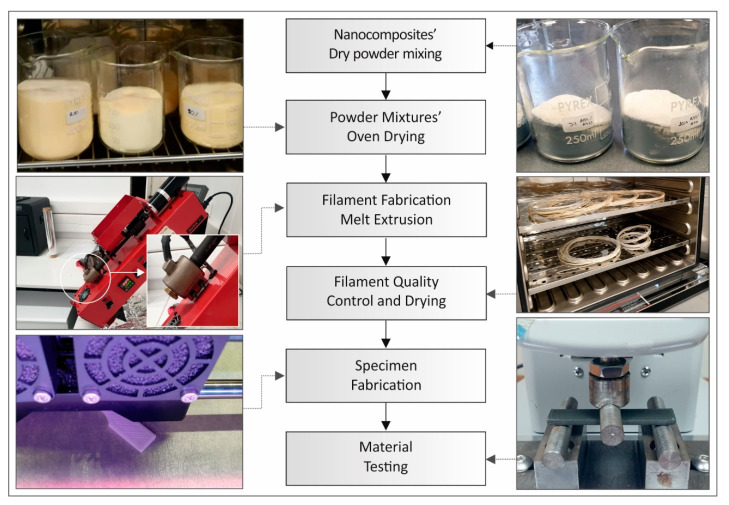
Nanocomposite filaments’ and specimens’ fabrication methodology.

Initially, the ABS matrix material was physically mixed with TiO_2_ and ATO fillers via a mechanical homogenizer. The result from this procedure was a homogenized powder mixture with different amounts of TiO_2_ (0.5, 2.5, 5, and 10 wt%). The same procedure was followed for the ABS/ATO, while both are summarized and depicted in Figure 1.

The powder mixtures were then dried in a laboratory oven, at 70 °C for 48 h before the extrusion process. The powder mixtures where fed into a Noztek Pro (Shoreham, UK) single screw extruder, preheated at 350 °C, a temperature determined experimentally.

Samples from the nanocomposites’ filaments were initially analyzed before being used for 3D printing. All specimens were 3D printed in the horizontal orientation (Figure 1). Specimens were then tested for the determination of their mechanical properties at room temperature of 23 °C.

The commercially available desktop 3D printer MakerBot (NY, USA), model replicator 2x was used in all cases. The specimens were built with the following 3D printing parameters: 100% solid infill, 45 degrees deposition orientation angle, 0.2 mm layer height and 235 °C 3D printing nozzle temperature.

### 2.3. Compositional and Structural Characterization Methods

Detailed scanning electron microscopy (SEM) characterization of the nanocomposites’ microstructure was carried out using a Nova NanoSEM 630 field emission SEM (FE-SEM, FEI Company, USA), equipped with an EDX detector (EDAX TEAM™, USA). In order to perform the SEM investigations, samples were coated first by thermal evaporation with a 5-nm Au thin film to avoid charging effects during the analyses.

The particle size and shape were studied using atomic force microscopy in tapping mode (TM-AFM). AFM images (height data) were recorded with a scanning probe microscope (MicroscopeSolver P47H Pro, NT-MDT, Moscow, Russia) in air at room atmosphere and temperature of 23 °C at a resonant frequency of about 300 kHz. Commercially available silicon cantilevers were used with scanning frequency of 1 Hz, a tip cone angle of 20°, a cantilever spring constant of 35 N/m, and tip radius of about 10 nm. Particle size/diameter evaluation was performed after a second flattening operation over the captured area of 2 × 2 μm^2^ height images and further grain analysis function using the Nova RC-1 NT-MDT image analysis software. The average size of both TiO_2_ and ATO NPs was statistically extracted from the corresponding AFM images by measuring the size of 100 single particles, while the mean value and the standard deviation were further calculated and reported.

Raman spectra were measured with a Labram HR-Horiba (Kyoto, Japan) scientific micro-Raman system. The spectra were carried out in the back-scattering geometry, while 514.5 nm line of an Ar^+^ ion laser operating at 1.5 mW power at the focal plane was utilized for the Raman excitation. An optical microscope equipped with a 50× long working distance objective has been utilized both for delivering the excitation light and collecting the back-scattering Raman activity.

### 2.4. Thermal Properties

Thermogravimetric analysis (TGA) was performed under nitrogen atmosphere to obtain information about the working and degradation temperature of the ABS matrix selected herein as well as the fabricated ABS nanocomposites. The measurements were carried out with a Perkin Elmer Diamond TG/DTA (Waltham, MA, USA) instrument at a temperature range varying from ambient (25 °C) up to 600 °C with a heating rate of 10K/min.

### 2.5. Tensile Tests

Tensile specimens were 3D printed as specified in the ASTM D638-02a standard (type V specimens with 3.2 mm thickness) [34]. For each case studied, seven 7 specimens were manufactured, in accordance to the ASTM D638-02a standard, which requires at least 5 specimens to be tested for each case. The tensile tests were performed using an Imada MX2 (Northbrook, IL, USA) tensile test apparatus, equipped with standardized grips. The chuck of the tensile test machine was set at a 10 mm/min speed for testing. This apparatus tenses the specimen fixed within and effectively and accurately measures and logs it in a file at 2000 Hz sampling rate, force (Newton) versus displacement (mm) experimental data in the specimen until it breaks.

### 2.6. Flexural Tests

Flexural test specimens were manufactured, according to ASTM D790-10 (64 mm length, 12.4 mm width, and 3.2 mm thickness) [35]. Seven specimens were 3D printed for each case studied, in accordance to with the ASTM D790-10 standard, which requires a minimum of 5 specimens to be tested for each case. The flexural tests were performed using an Imada MX2 (Northbrook, IL, USA) tensile test machine, with a setup compatible to the standard (three-point bending test with 52 mm support span). The chuck of the tensile test machine was set at a 10 mm/min speed for testing. Force (Newton) versus displacement (mm) experimental data are logged in a file at 2000 Hz sampling rate.

### 2.7. Micro-Hardness Tests

Micro-hardness tests were performed according to the specifications of the ASTM E384-17 standard [36]. The micro-Vickers method was applied, with 0.2 kg force scale (1.962 N) and 10 s indentation time. A typical 136° apex angle Vickers diamond pyramid was used as indenter. This indenter was forced onto a polished surface of the specimens. After the diamond pyramid is removed, the remaining imprint’s mean diagonals are employed, for the calculation of the area of the remaining indentation. This is an automated process, with the calculations performed directly by the apparatus. Experiments were carried out with an Innova Test 400-Vickers (Maastricht, The Netherlands) apparatus.

## 3. Results

### 3.1. Experimental Observations Regarding Filament and Specimens’ Fabrication

During the experimental efforts and protocols followed to develop the ABS/TiO_2_ and ABS/ATO materials, filaments, and specimens, several significant experimental observations were made. Filaments that were not appropriately dried (70 °C, 48 h), led to 3D printed structures with inferior properties and often to clogged 3D print heads. Another observation was that the reduction of printing speed to 20 mm/s and the increase of material flow to 110%, resulted in more consistent, quality printed structures.

In order to avoid applying 3D printing adhesive aids (such as printing glues), as well as to save the building specimens from warping or detaching from the heated print bed, it was observed that it is essential to have a fully closed 3D printing area in order to maintain constant temperature in the 3D printing chamber.

### 3.2. Microstructure Characterization

**Figure 2:**
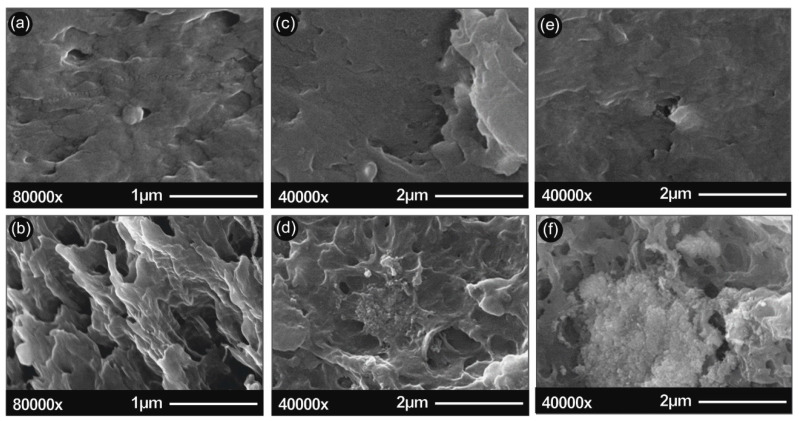
SEM images of (**a**) ABS surface; (**b**) ABS section area; (**c**) ABS/TiO_2_ 5 wt% surface; (**d**) ABS/TiO_2_ 5 wt% section area; (**e**) ABS/ATO 5 wt% surface; (**f**) ABS/ATO 5 wt% section area.

In Figure 2, the SEM images of the 3D printed samples surface as well as the cross-sectional fracture surface are given for unfilled/ neat ABS, ABS/TiO_2_ and ABS/ATO nanocomposites. Namely the surface microstructures of (a) ABS surface, (b) ABS section area, (c) ABS/TiO_2_ 5 wt% surface, (d)ABS/TiO_2_ 5 wt% section area, (e) ABS/ATO 5 wt% surface and (f) ABS/ATO 5 wt% section area are illustrated.

### 3.3. AFM Analysis of TiO_2_ and ATO Nnanoparticles’ Size

**Figure 3:**
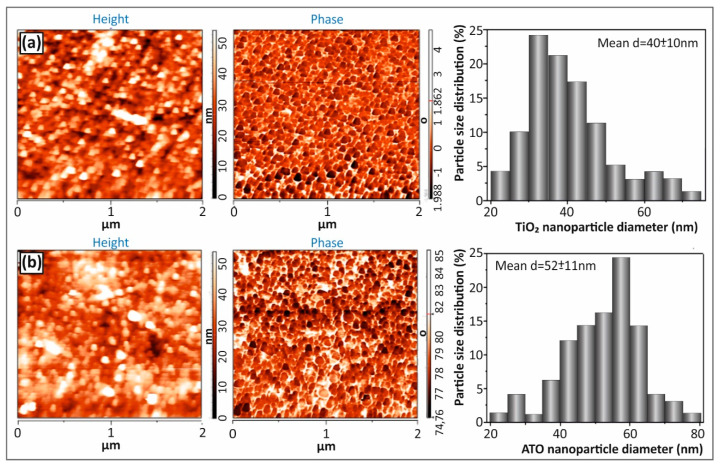
AFM height and phase images of (**a**) TiO_2_ and (**b**) ATO NPs showing the particle spherical morphologies (phase images) and average particle diameters calculated from the corresponding height images representative of the image topography. 100 NPs were measured from each AFM height image to extract the average diameter and standard deviation values (scan size: 2 × 2 μm^2^).

Figure 3 illustrates the captured AFM height and phase images of (a) TiO_2_ and (b) ATO spherical NPs at a scanned area of 2 × 2 μm^2^ together with the corresponding histogram of the particle size distribution (the value of the particle mean diameter and the standard deviation is given for both cases as inset in the corresponding histogram). Specifically, the spherical NP morphology is revealed by the phase images, while the average particle diameters have been calculated from the respective height images. Namely, TiO_2_ NPs exhibit a diameter of 40 ± 10 nm, while ATO NPs 52 ± 11 nm, being in good agreement with the supplier’s data sheet specifications regarding the average particle size. It should be mentioned that 100 NPs were measured from each AFM corresponding height image to extract the average diameter and standard deviation values.

### 3.4. Raman Analysis

**Figure 4:**
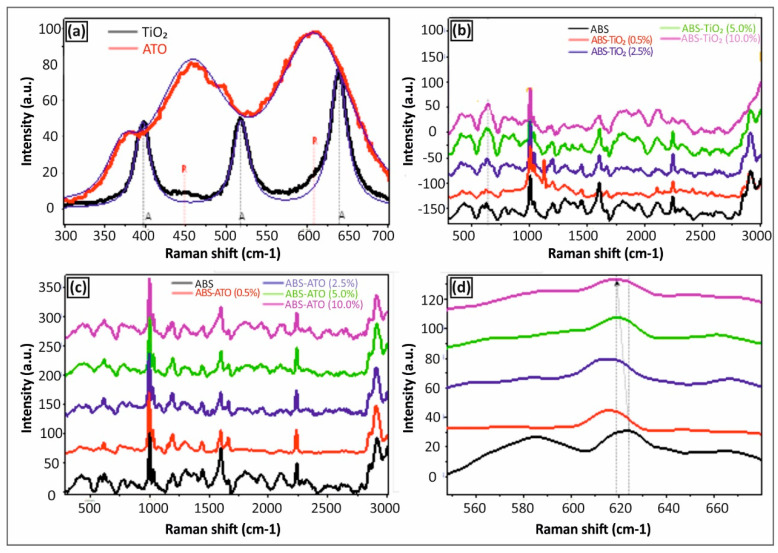
Raman spectra of (**a**) TiO_2_ and ATO NPs in the form of films prepared by drop casting, (**b**) ABS/TiO_2_ nanocomposites, and (**c**,**d**) ABS/ATO nanocomposites with different particle loadings.

Figure 4 illustrates the Raman spectra of (a) TiO_2_ and ATO NPs in the form of films prepared by drop casting, as well as (b) ABS/TiO_2_ nanocomposites, and (c,d) ABS/ATO nanocomposites at different particle loadings.

### 3.5. Thermal Analysis

**Figure 5:**
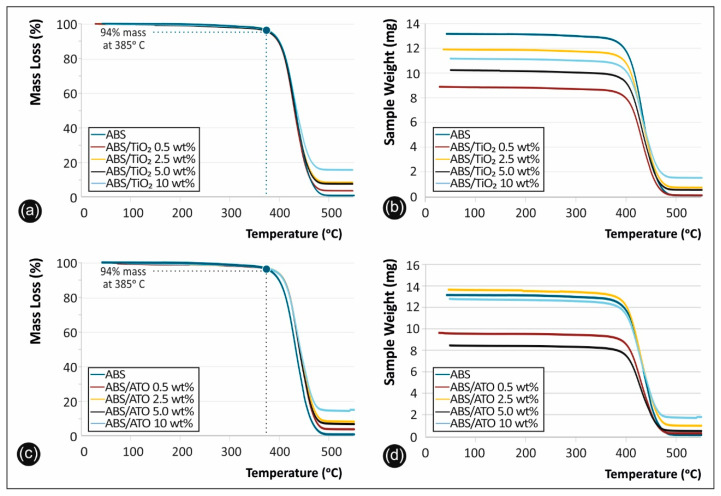
Experimentally determined TGA mass loss (wt%) and sample weight (mg), versus temperature curves for the neat ABS Terluran Hi-10 and all the nanocomposites fabricated in this study: (**a**,**b**) refer to the ABS/TiO_2_ nanocomposites, while (**c**,**d**) refer to the ABS/ATO nanocomposites of the studt.

The TGA scans of the mass loss (%) versus temperature and the sample weight (mg) versus temperature for the neat ABS polymer matrix, as well as the ABS/TiO_2_ (Figure 5a,b) and ABS/ATO (Figure 5c,d) nanocomposites are summarized in Figure 5.

### 3.6. Tensile Properties

**Figure 6:**
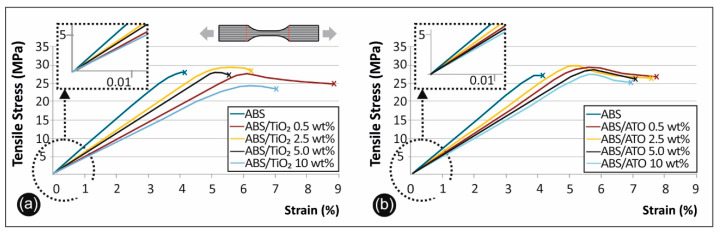
(**a**) Tensile stress vs. strain graphs for ABS/TiO_2_ and (**b**) ABS/ATO.

**Figure 7:**
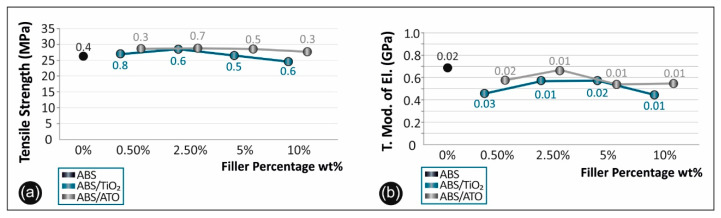
(**a**) Comparative tensile strength graph and (**b**) tensile mod. of elasticity for all the materials studied.

Figure 6 shows the stress–strain curves derived and calculated from the tensile testing of ABS/TiO_2_ (Figure 6a) and ABS/ATO (Figure 6b). Figure 7a summarizes and compares the tensile strength versus the filler percentage of the unfilled ABS, the ABS/TiO_2_, and the ABS/ATO, while Figure 7b, summarizes and compares the tensile modulus of elasticity versus the filler percentage of the unfilled ABS, the ABS/TiO_2_, and the ABS/ATO nanocomposites for the various concentrations studied.

### 3.7. Flexural Properties

**Figure 8:**
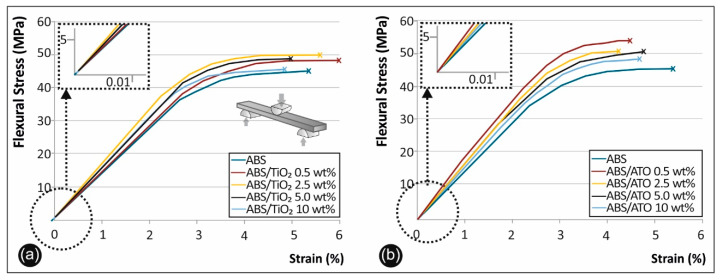
(**a**) Flexure stress vs. strain graphs for (**b**) ABS/TiO_2_ and ABS/ATO.

Figure 8 shows the stress–strain curves derived and calculated from the flexural testing of ABS/TiO_2_ (Figure 8a) and ABS/ATO (Figure 8b) in respect to pure ABS.

**Figure 9:**
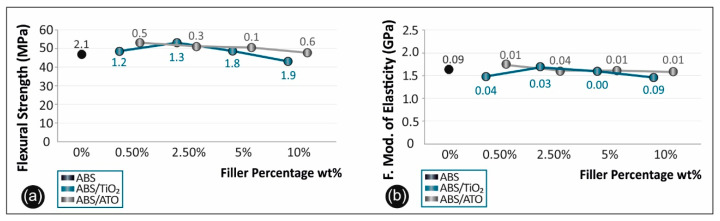
(**a**) Comparative flexural strength graph and (**b**) flexural mod. of elasticity for all the materials studied.

In Figure 9a, the flexural strength versus the filler percentage of the unfilled ABS, the ABS/TiO_2_ and the ABS/ATO, as well as in Figure 9b the flexural modulus of elasticity results for the aforementioned materials, are summarized and compared.

### 3.8. Micro-Hardness Results

**Figure 10:**
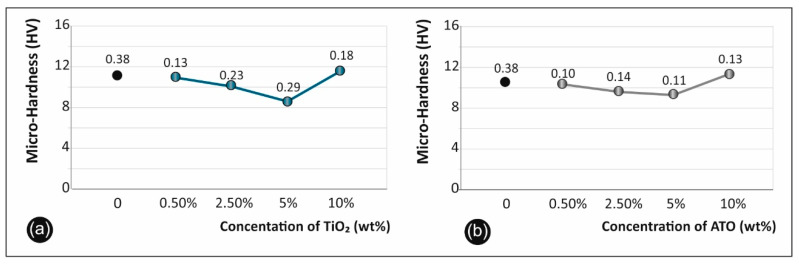
Micro-Hardness Vickers results of ABS/TiO_2_ (**a**) and ABS/ATO (**b**) versus the filler concentration.

Regarding the micro-hardness testing of the materials studied, the results are summarized in Figure 10a for the cases of ABS/TiO_2_ and in Figure 10b for the cases of ABS/ATO, for the various concentrations studied.

## 4. Discussion

### 4.1. Structural and Compositional Characterization

SEM characterization was used to analyze the nano/micro structuring of both the surface of printed material, as well as the fracture area of the 3D printed filaments. In Figure 2, typical images of the 3D printed material (surface and section) for pure ABS and the nanocomposites studied with 5% TiO_2_ or ATO filler are presented.

As can be seen from the above figures, low filler content leads to a more elastic fracture, with more polymer spikes on the fracture surface when compared to pure ABS. In contrast, above 5% filler content, in both ABS/TiO_2_ and ABS/ATO nanocomposites, filler agglomeration is visible. These agglomerations, seem to promote a more brittle fracture behavior, promoting a “cleaner” fracture surface when compared to lower filler concentrations. These results agree with the tensile stress graphs in Figure 6a,b. Also, it was evident that in the filament and the specimens under 5% filler loading, there were no air bubbles present in the nanocomposites structure, thus leading to the assumption that the extrusion produced nanocomposites with no internal structuring faults.

In the Supplementary material of the manuscript, detailed SEM investigations of the ABS/TiO_2_ (Figure S1) and ABS/ATO (Figure S2) nanocomposite fractured surfaces at 0.5 wt%, 2.5 wt%, 5 wt% and 10 wt% are presented at three different magnifications for each filler loading. The main findings of the SEM analysis could be summarised as following: (i) no voids or discontinuities have been observed in the nanocomposite materials’ micro-structure indicating a high quality of melt-mixing process, (ii) typical amorphous morphology of a polymer has been observed for all ABS nanocomposites, (iii) no visible particle micro-aggregates could be seen for the 0.5 wt% and 2.5 wt% ABS/TiO_2_ and ABS/ATO nanocomposites, respectively, and (iv) by increasing the filler content to 5 wt% and 10 wt%, some micro-aggregates distributed throughout the whole nanocomposite fractured surface could be observed, with a larger number of micro-aggregates and the aggregation effect to be more prominent for the 10 wt% nanocomposites. Moreover, in the Supporting Information file, the SEM images of TiO_2_ and ATO nanoparticles at two different magnifications are depicted (Figure S3), indicating spherical nanograined crystals for both TiO_2_ and ATO nanoparticles. Finally, representative EDX spectra of the TiO_2_ and ATO nanoparticles (Figure S6), as well as the ABS/TiO_2_ (Figure S4) and ABS/ATO (Figure S5) nanocomposites at 0.5 wt%, 2.5 wt%, 5 wt%, and 10 wt% are presented, verifying the main chemical elements of each material formulation.

### 4.2. AFM Nanoparticle Size Analysis

As it can be observed by the corresponding AFM images (Figure 3) and the performed statistical analysis, the TiO_2_ and ATO NPs are in the range of 50 nm with a relatively good mondispersity and without any observed large particles. Namely, the TiO_2_ NPs exhibited diameters of 40 ± 10 nm, while the ATO NPs were found to be slightly bigger in size, i.e., 52 ± 11 nm. The monodispersed commercially available TiO_2_ and ATO NPs used in this work are envisaged to be ideal reinforcement candidates for a polymer matrix as for instance the ABS in our study that can effectively endow mechanical reinforcement and any inherent physical property to the final nanocomposites arising from the incorporated respective NPs.

### 4.3. Raman Analysis

Raman spectroscopy was applied as a characterization method to study the occurring phases of TiO2 and Sb-doped SnO_2_ (ATO) NP thick films created by drop casting of pre-dispersed nanoparticle solutions on silicon wafer substrates. Figure 4a indicates the Raman spectra in the range of 300–700 cm^−1^ for both the TiO_2_ and ATO NPs. For the TiO_2_ NPs, the anatase and the rutile phase have different Raman active modes. The anatase main peaks are located at 395 (B_1g_), 513 (A_1g_), and 639 cm^−1^ (E_g_), and the rutile peaks at 447 and 605 cm^−1^, respectively [37]. Specifically, for the commercial TiO_2_ NPs studied herein, it can be speculated that the TiO_2_ NPs consist mainly of anatase phase with three strong peaks at 395 (B_1g_), 513 (A_1g_) and 639 cm^−1^ (E_g_). However, a small portion and existence of rutile phase can be observed appearing as very tiny shoulder peaks at 447 and 605 cm^−-1^, respectively. The Raman spectra of ATO NPs showed bands centered at 358, 474 (E_g_), 632 (A_1g_) cm^−1^ being in good agreement with the observed characteristics of Sb doped SnO_2_ nanowires reported by Costa et. al. [38]. These results show the typical feature of the rutile phase of SnO_2_ NPs with the additional vibrational mode centered the 358 cm^−1^ attributed to the Sb dopant of the SnO_2_ crystal. The inactive mode at 358 cm^−1^ comes from the condition arisen by the antimony incorporation into the SnO_2_ structure [39]. However, it is important to state that this explanation is the most possible presented in the literature, so far. Another possibility is the segregation of Sb atoms toward the surface, which was recently reported by Stroppa et al., where the dependence of the surface energy on the Sb doping level was observed [39].

Figure 4b shows the Raman spectra of ABS/TiO_2_ 3D printed nanocomposites at different particle loadings in the spectral region of 300–3000 cm^−1^. For all nanocomposites, the characteristic ABS Raman peaks could be seen typical for the vibrations of the acrylonitrile butadiene styrene copolymer as have been reported elsewhere [40]. It is worth mentioning that the peak at ~640 cm^−1^ of the TiO_2_ nanocrystallites is evident for the nanocomposites with loadings > 5.0 wt% with the TiO_2_ anatase peak being very apparent and visible at the 10 wt% filler loading.

Figure 4c,d show the Raman spectra of ABS/ATO 3D printed nanocomposites at different particle loadings in the spectral region 300–3000 cm^−1^ and 540–700 cm^−1^, respectively. For all nanocomposites and even for the highest loaded sample of 10 wt%, the nanoparticulate crystallites responsible for the active vibration modes in Raman as observed and shown in Figure 4a have not been detected from all the investigated blended ABS/ATO nanocomposite systems. However, an interesting shift of an ABS peak at ca. 610 cm^−1^ to lower frequencies is an indirect proof of the blended ATO NPs in the polymer matrix, which is attributed to the 610 cm^−1^ active vibration mode of ATO NPs.

### 4.4. Thermal Analysis

Dynamic thermogravimetric scans helped to elucidate the thermal stabilities of the ABS/TiO_2_ and ABS/ATO nanocomposites fabricated in this study as novel and high-performance 3D printable engineered thermoplastic materials. From the TGA analysis, it was shown that the working temperature utilised in this study for both ABS filament production via compounding and extrusion process, as well as the 3D printing manufacturing process are below the critical temperature of 385 °C, where ABS starts degrading rapidly accompanied with an abrupt weight loss. Both ABS/TiO_2_ and ABS/ATO nanocomposites lost 6% weight (T_d_^6%^) at 385 °C, while the whole ABS decomposition process occurred then up to ~500 °C, with the remnant material above 500 °C in all cases corresponding to the inorganic TiO_2_ and ATO nanocrystal inclusions in the respective nanocomposites.

### 4.5. Tensile Test Results

Regarding the tensile strength of ABS/TiO_2_ nanocomposites, depicted in Figure 6a, there is an increase of 7% at the 2.50 wt% TiO_2_ filler concentration, when compared to pure ABS specimens. On the other hand, the tensile modulus of elasticity has a peak value of 687 MPa in the case of pure ABS (Figure 7a). Similar research regarding printed PA6/TiO_2_ nanocomposites reported an increase in the tensile strength by 21.31% at 30 wt% filler concentration, when compared to virgin material [41,42]. Another research by Torrado et al. [24] reported similar values but with no reduction or improvement in the tensile strength regarding the 3D printed ABS/TiO_2_ 5 wt% nanocomposites when compared to virgin ABS [43]. Regarding the modulus of elasticity, it increases for concentrations up to 5 wt% and rapidly decreases in higher concentrations, as it is shown in Figure 7b.

Regarding the tensile strength of ABS/ATO nanocomposites, depicted in Figure 7b, there is an increase of 9.2% at 0.50 wt% filler concentration when compared to pure ABS. Moreover, the modulus of elasticity, it increases for concentrations up to 2.5 wt% and rapidly decreases in higher concentrations, as it is shown in Figure 7b and has again the highest value in the case of pure ABS (Figure 7b). For the ABS/ATO 3D printed nanocomposites, there is limited to no literature available to compare the overall results with.

On the other hand, there are several possible mechanisms that might lead to an increase in the material strength, since the nanoparticles agglomerate, and one of them is their interaction with the polymer matrix [4,43]. The effective size of the filler plays an important role in determining the mechanical properties of the final composite. There is a general rule that, as the size of the filler particles decrease, the effective filler surface area increases along with the interactions with the matrix. Moreover, at higher filler loadings, the polymer chains become immobilized, while there is a significant concentration of stress upon the points of agglomeration [4]. This induces fracture points [44] and thus hampers the mechanical properties of the composite. The increase found in the current research in the tensile strength and in the tensile modulus of elasticity (up to specific concentrations for each nano material, as described) of the nanocomposites studied herein confirms the above scenario.

### 4.6. Flexural Test Results

Flexural test results on the ABS/TiO_2_ specimens showed a peak value of 52.50 MPa in the case of 2.50% filler percentage, with an overall 12% increase when compared to pure ABS (Figure 8a). Flexural modulus of elasticity in this case, has a peak value of 1676.11 MPa also at 2.50% filler concentration (Figure 9a).

Moreover, regarding the ABS/ATO specimens, the flexure strength max peak value appears in 0.50 wt% filler concentration with an overall increase of 13% when compared to pure ABS (Figure 8b). About the flexural modulus of elasticity, the max value appeared again in the case of 0.50 wt% filler concentration with an increase of 6.8% compared to pure ABS (Figure 9b).

Similar results were reported in the literature about ABS/TiO_2_ nanocomposites, where there is a peak value of 72 MP at 5% and 10 wt% filler concentrations [4]. The 1 wt%. TiO_2_ nanocomposites studied by Skorski et al. showed a decrease in both ultimate tensile strength (UTS) and flexural strength as compared to the 0% sample. The 5 and 10 wt% TiO_2_ nanocomposites show an increase in UTS and flexural strength in comparison to the 0% and 1% samples [4].

### 4.7. Micro-Hardness Results

The results on the micro-hardness regarding the ABS/TiO_2_ specimens showed a peak value at 10 wt% filler concentration. The overall increase when compared to pure ABS specimens is 5.9% (Figure 10a). The results regarding the ABS/ATO specimens show a max value at 10% filler concentration, with an overall increase of 6% (Figure 10b). The increase in microhardness, especially after 10 wt% concentrations is caused due to structuring alterations that the fillers induced in the ABS matrix. A similar trend is showed in literature [45] when comparing ABS/ZnO nano and microcomposites to pure ABS. More specifically, it was evident that, overall, ABS/ZnO nanocomposites had the higher micro-hardness values, when compared to pure ABS and to ABS/ZnO microcomposites. At 0.5 wt% concentration, both showed increased micro-hardness when compared to pure ABS. Both composites’ hardness kept reducing till 5 wt% filler concentration, while after 5 wt% micro-hardness kept rapidly increasing, reaching the peak value at 20 wt% filler concentration. Furthermore, regarding micro-hardness in 3D printed specimens, the literature is limited to non-nanocomposite materials.

## 5. Conclusions

**Figure 11:**
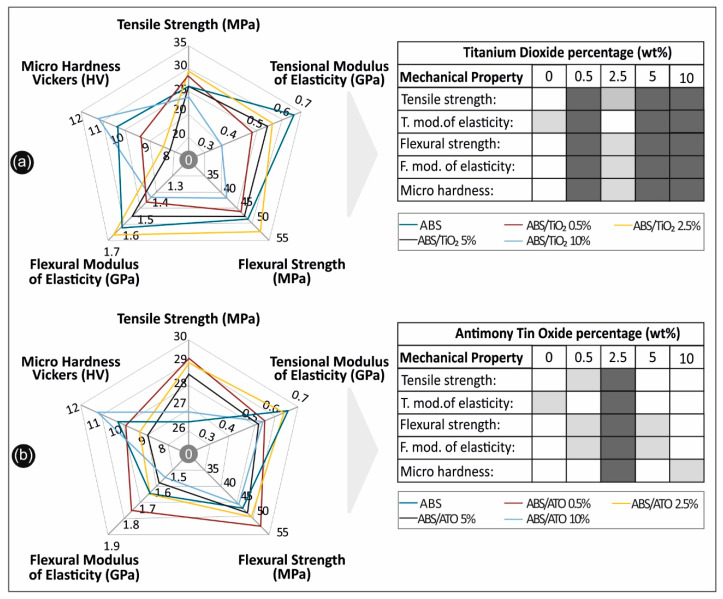
Overall comparative graphs for (**a**) ABS/TiO_2_ nanocomposites and (**b**) ABS/ATO, in all scenarios studied.

In this study, novel nanocomposite filaments comprising of ABS/TiO_2_ and ABS/ATO were developed in various concentrations for application in fused filament fabrication (FFF), an approach not presented in the literature so far in terms of the nano fillers used with this specific polymer matrix and the methodology for the implementation of the study. When comparing to previous studies with solvent based methods [46,47], it was proven that, with the fabrication methodology described herein, it is possible to fabricate physically and mechanically improved, more homogeneous nano or micro composite materials for direct commercial or industrial implementation, while incorporating a viable, clean (solvent-free), commercially applicable methodology. From the overall assessment of the results, which are summarized in Figure 11, it can be concluded that the implementation of nano fillers can not only alter the mechanical and physical response of a polymer matrix, but also noticeably improve it. Such nanocomposites with improved mechanical properties can be easily manufactured and utilized in various industrial applications related to the unique specifications of these nano fillers, which are combined in this work with a popular polymer matrix.

## Supplementary Materials

The following are available online at https://www.mdpi.com/2073-4360/12/7/1589/s1, Figure S1: SEM of (a–c) ABS/TiO_2_ 0.5% fracture area, (d–f) ABS/TiO_2_ 2.5% fracture area, (g–i) ABS/TiO_2_ 5% fracture area, (j–l) ABS/TiO_2_ 10% fracture area, Figure S2: SEM of (a–c) ABS/ATO 0.5% fracture area, (d,e) ABS/ATO 2.5% fracture area, (g–i) ABS/ATO 5% fracture area, (j–l) ABS/ATO 10% fracture area, Figure S3: SEM of (a,b) TiO2 nano powder and (c,d) ATO nano powder, Figure S4: EDX spectra of (a) ABS/TiO_2_ 0.5%, (b) ABS/TiO_2_ 2.5%, (c) ABS/TiO_2_ 5%, (d) ABS/TiO2 10%, Figure S5: EDX spectra of (a) ABS/ATO 0.5%, (b) ABS/ATO 2.5%, (c) ABS/ATO 5%, (d) ABS/ATO 10%, Figure S6: EDX spectra of (a) TiO_2_ nano powder and (b) ATO nano powder.
