# Supplementary material for: Mechanical Properties of 3D-Printed Acrylonitrile–Butadiene–Styrene TiO2 and ATO Nanocomposites
Source: Polymers (Basel). 2020 Jul 17;12(7):1589. doi: 10.3390/polym12071589 (PMC7407130; doi:10.3390/polym12071589)
Supplement: Supplementary file 1 [file polymers-12-01589-s001.pdf]

Title: Mechanical properties of 3D-Printed Acrylonitrile–Butadiene–Styrene TiO_2 and ATO nanocomposites

Journal: Polymers

Supplementary Material

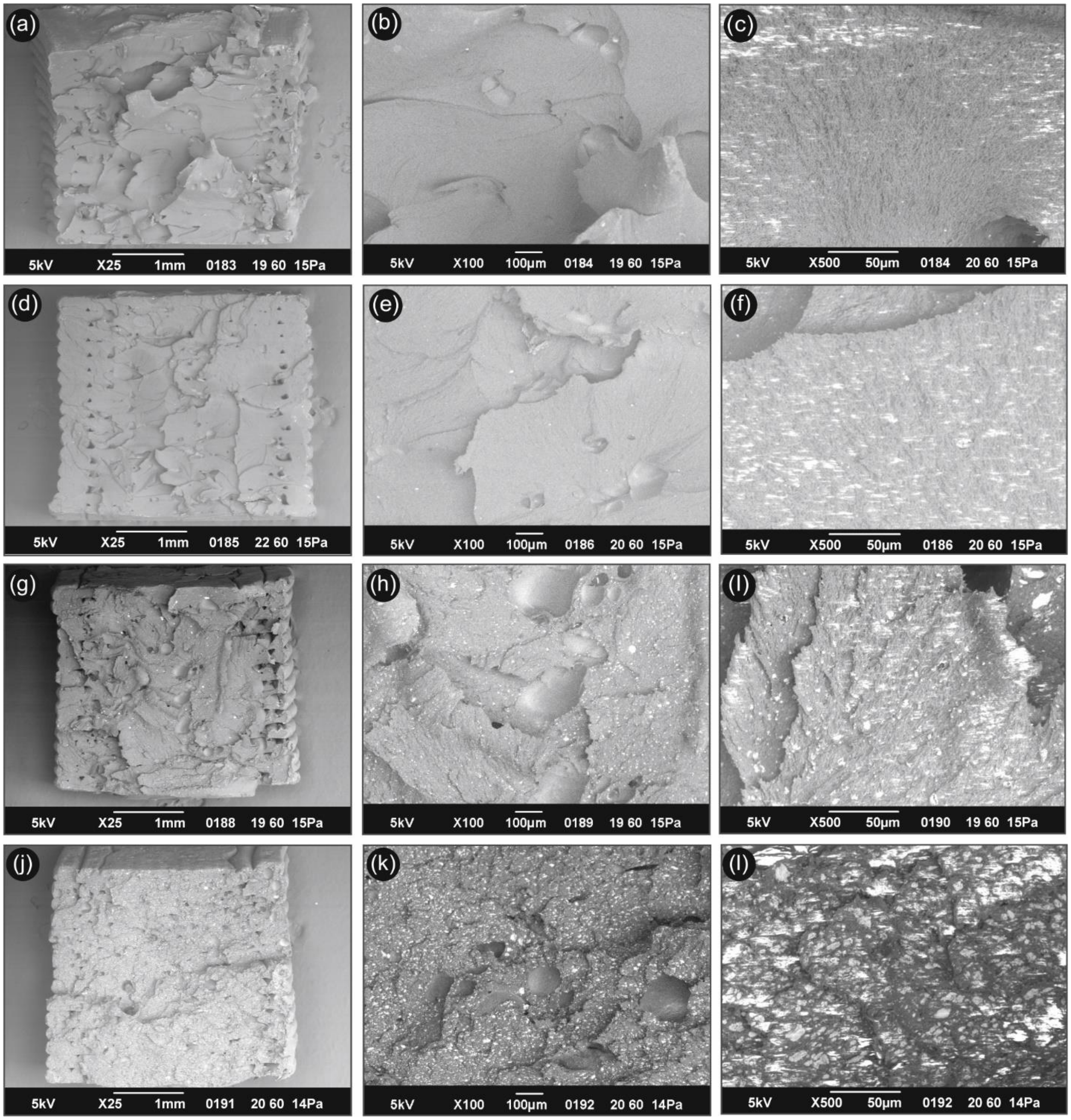

Figure S1: SEM of (a-c) ABS/TiO_2 0.5% fracture area, (d-f) ABS/TiO_2 2.5% fracture area, (g-i) ABS/TiO_2 5% fracture area, (j-l) ABS/TiO_2 10% fracture area

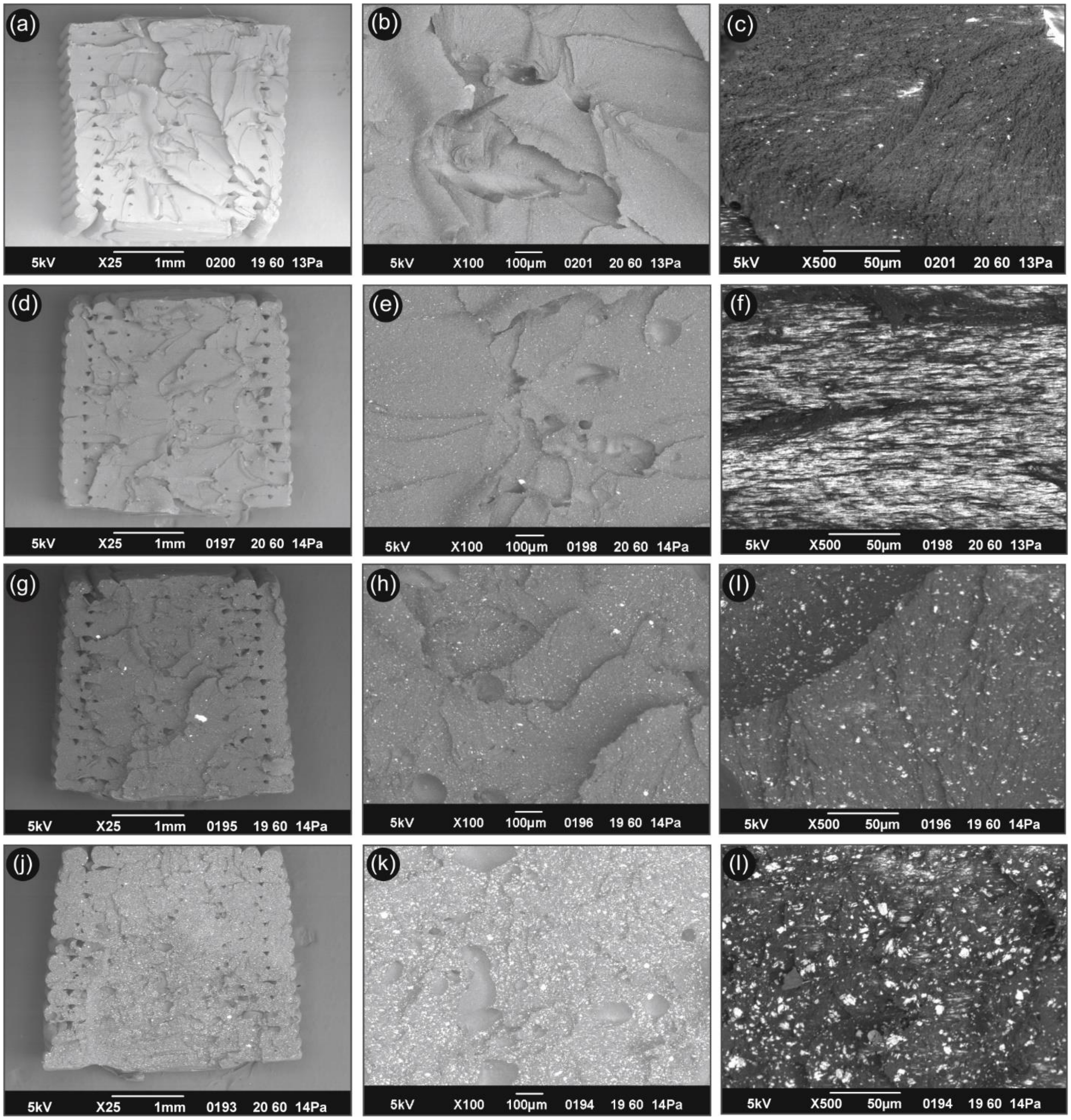

Figure S2: SEM of (a-c) ABS/ATO 0.5% fracture area, (d-e) ABS/ATO 2.5% fracture area, (g-i) ABS/ATO 5% fracture area, (j-l) ABS/ATO 10% fracture area

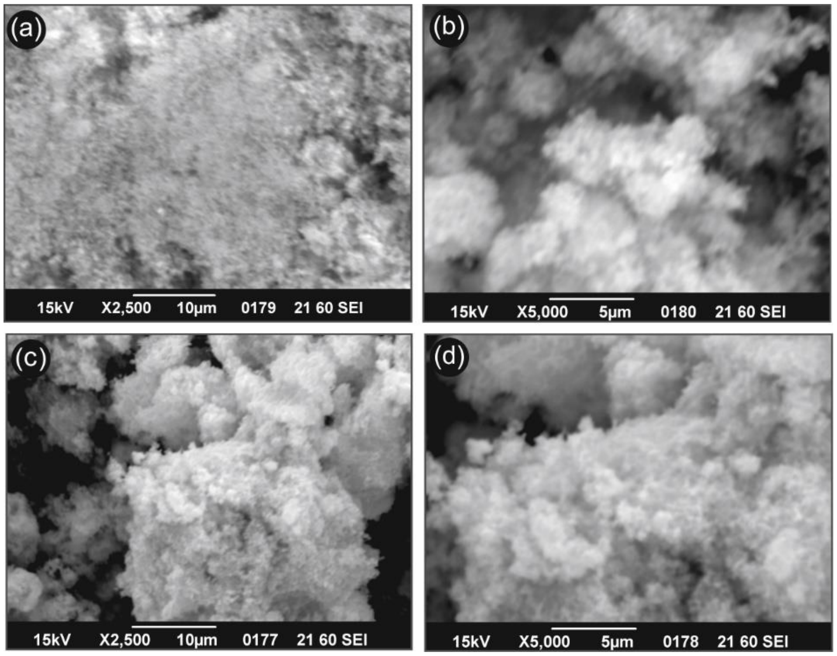

Figure S3: SEM of (a-b) TiO_2 nano powder and (c-d) ATO nano powder

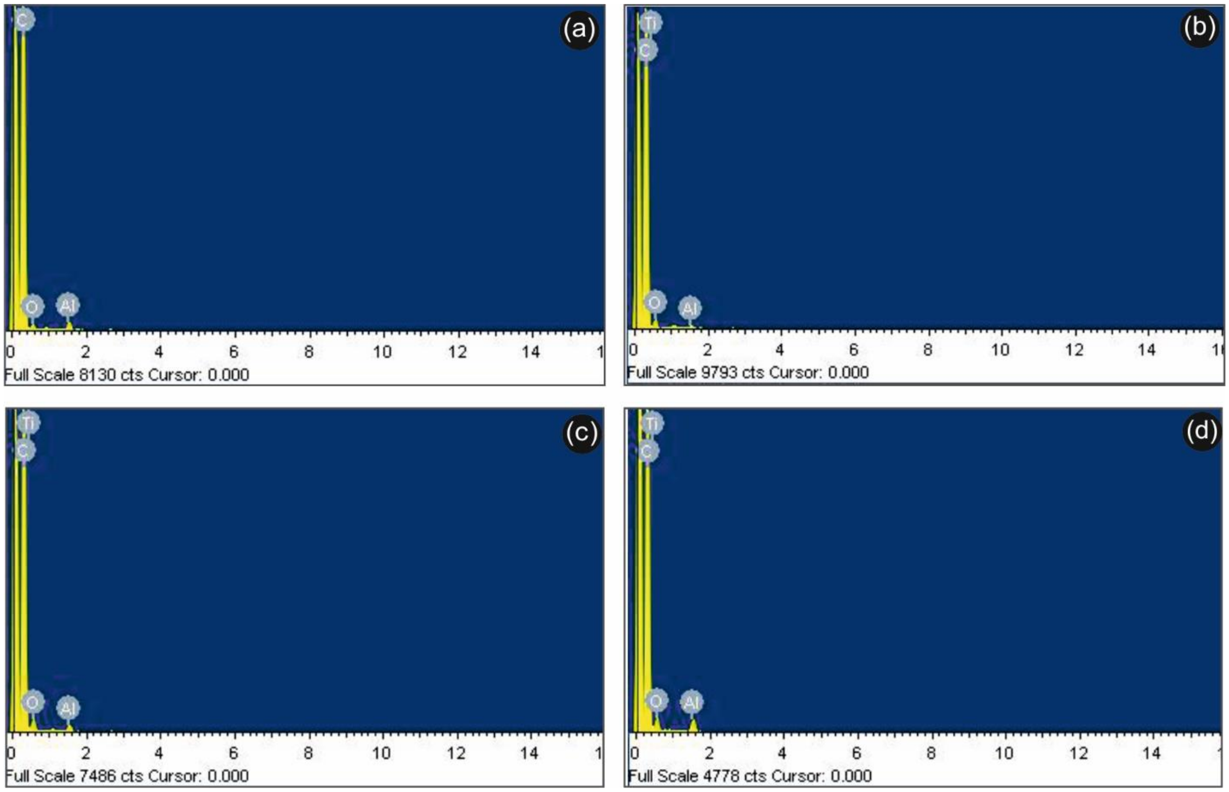

Figure S4: EDX spectra of (a) ABS/TiO_2 0.5%, (b) ABS/TiO_2 2.5%, (c) ABS/TiO_2 5%, (d) ABS/TiO_2 10%

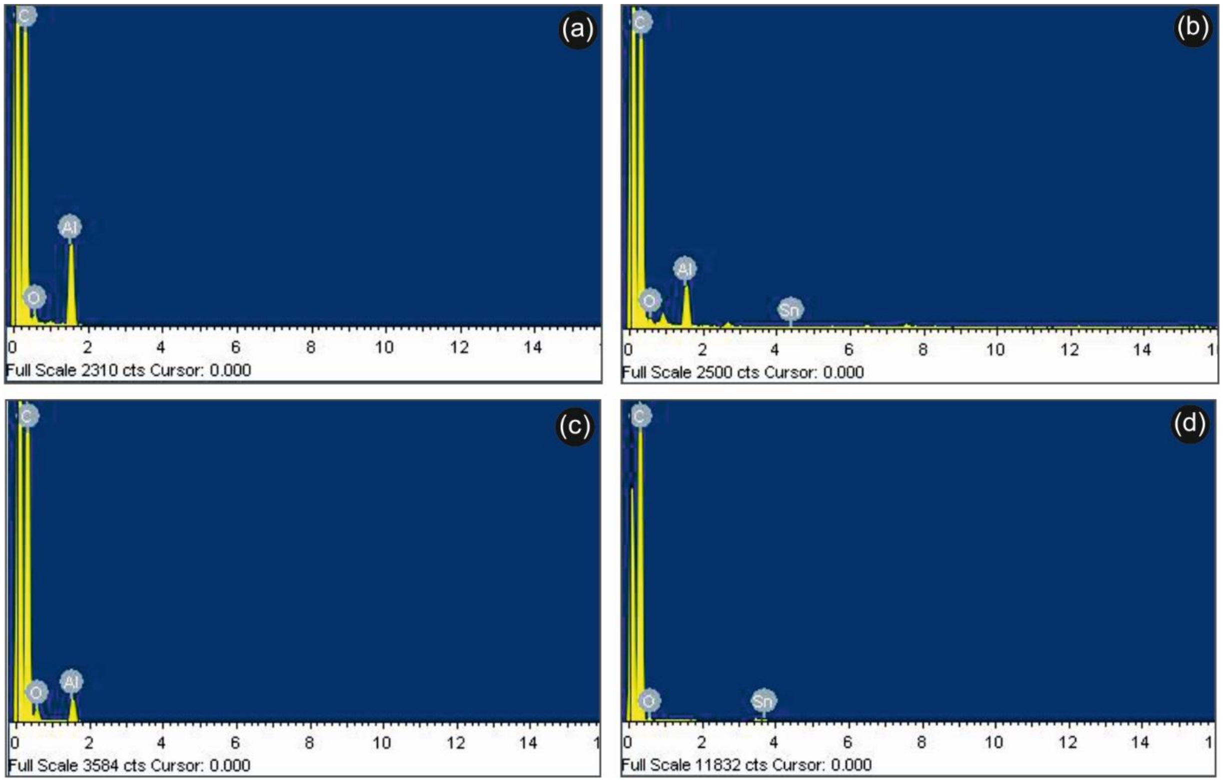

Figure S5: EDX spectra of (a) ABS/ATO 0.5%, (b) ABS/ATO 2.5%, (c) ABS/ATO 5%, (d) ABS/ATO 10%

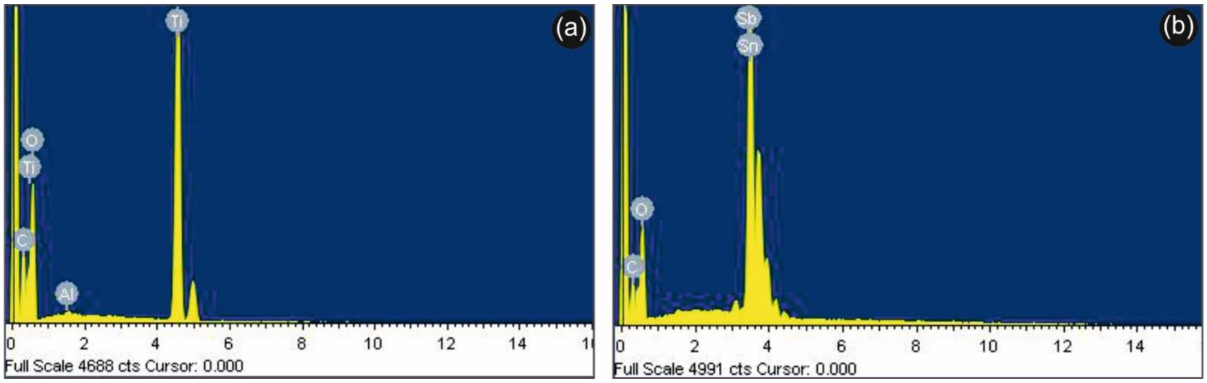

Figure S6: EDX spectra of (a) TiO_2 nano powder and (b) ATO nano powder

Note: SEM images were taken on a JEOL JSM 6390LV electron microscope in low vacuum mode at 5kV and 15kV acceleration voltage on non-coated samples. The SEM fractured surfaces has been observed that with the increase of the filler content develop a slightly higher rough surface due to the filler amount that is possible to start creating microaggregates at higher than 2 wt.% content.

Note: EDX Spectra were taken on a Oxford instruments INCA x-act in high vacuum mode on non-coated samples.
